# The Best Period of Operating for Laceration of the Perineum

**Published:** 1871-09

**Authors:** 


### The Best Period of Operating for Laceration of the Perineum.

Discussion in the Gynæcological Society of Boston.

Dr. Wheeler desired to ascertain the opinion of members of the Society as to the best period of operating for laceration of the perineum. He had recently seen an extract of a few lines from a paper contributed to the *Glasgow Medical Journal* for November, 1869, by Dr. John Brinton, Surgeon to the Royal Maternity Charity, London, in which he advocates operating immediately after delivery. He narrates three cases so treated, and remarks:

"1. That the result of the recent operation is very satisfactory.

"2. That the operation is very easy, and,

"3. That it is comparatively free from danger, and is nearly painless, requiring no chloroform; because the parts which have been torn are in an anæsthetic state, being benumbed by the pressure they have recently undergone."

What, Dr. Wheeler would ask, was the usual practice in Boston?

Dr. Martin replied that it was usual to wait till a subsequent period. He had never, however, been satisfied with the reasons ordinarily given for this delay. To say, for instance, that the lochia would produce irritation at the line of suture, was but a trivial excuse. Dr. M. related several cases where he had operated immediately. In one he had delayed twenty-four hours, and yet obtained a perfect success, the new perineum sufficing for its work in several successive confinements.

Dr. Wheeler had first operated fifteen years ago. It had been, and was still, his habit to operate immediately after the occurrence of the accident. He thought that many of his neighbors did the same.

Dr. Storer believed that the frequency of perineal laceration, even to the sphincter ani, was greatly under-estimated. He seldom now saw it save in chronic cases, as he had long since relinquished even consultations in midwifery. He was satisfied, however, that the lesion occurred, at the least, ten times where it was detected once,—accouchers failing to examine the parts after delivery had been completed. Years afterwards, the patient might seek relief for prolapsus uteri, rectocele, etc., and the state of things then first become known. He frequently had to operate under such circumstances, and the result towards effecting a restoration of the patient to health was at times very marked.

Dr. Martin believed that the accident was often owing to the careless use of forceps. This was perhaps especial likely to occur in country practice, where consultations were often so much more difficult to obtain in cases of emergency. It was often owing, moreover, to carelessness during extraction of the fœtal shoulders. He had no doubt of the correctness of Dr. Storer's statement as to the lesion not being usually detected at the time of its occurrence. He had noticed that at a late meeting of the Obstetrical Society of this city, a gentleman who had practiced some three or four years, and had had "many cases," spoke as though they had occasioned him no subsequent difficulty. As far as that was concerned, many practitioners of real experience seem inclined not to attach any importance to the lesion.. They were all of them very wrong.

Dr. Weston had frequently heard similar remarks from veteran accouchers. He agreed with Dr. Martin, that it simply showed their ignorance of the after effects of perineal laceration, unremedied, upon a patient's health.

Dr. Martin believed that the usual method of supporting the perinium, previous to and during the passage of the fœtal head. directly predisposed to rupture.

Dr. Wheeler remarked that the reason why many physicians never found laceration of the perineum to occur in their own practice was that they never looked for it.—*Gynæcological Journal.*

